# High throughput detection of *Coxiella burnetii *by real-time PCR with internal control system and automated DNA preparation

**DOI:** 10.1186/1471-2180-8-77

**Published:** 2008-05-19

**Authors:** Marcus Panning, Jochen Kilwinski, Susanne Greiner-Fischer, Martin Peters, Stefanie Kramme, Dimitrios Frangoulidis, Hermann Meyer, Klaus Henning, Christian Drosten

**Affiliations:** 1 Clinical Virology, Bernhard-Nocht Institute for Tropical Medicine, Bernhard Nocht Str. 74, 20359 Hamburg, Germany; 2 Staatliches Veterinäruntersuchungsamt, Zur Taubeneiche 10-12, 59821 Arnsberg, Germany; 3 Bundeswehr Institute of Microbiology, Neuherbergstr. 11, 80937 Munich, Germany; 4 Friedrich-Löffler-Institut, Seestr. 55, 16868 Wusterhausen, Germany; 5 University of Bonn Medical Center, Sigmund-Freud-Str. 25, 53105 Bonn, Germany

## Abstract

**Background:**

*Coxiella burnetii *is the causative agent of Q-fever, a widespread zoonosis. Due to its high environmental stability and infectivity it is regarded as a category B biological weapon agent. In domestic animals infection remains either asymptomatic or presents as infertility or abortion. Clinical presentation in humans can range from mild flu-like illness to acute pneumonia and hepatitis. Endocarditis represents the most common form of chronic Q-fever. In humans serology is the gold standard for diagnosis but is inadequate for early case detection. In order to serve as a diagnostic tool in an eventual biological weapon attack or in local epidemics we developed a real-time 5'nuclease based PCR assay with an internal control system. To facilitate high-throughput an automated extraction procedure was evaluated.

**Results:**

To determine the minimum number of copies that are detectable at 95% chance probit analysis was used. Limit of detection in blood was 2,881 copies/ml [95%CI, 2,188–4,745 copies/ml] with a manual extraction procedure and 4,235 copies/ml [95%CI, 3,143–7,428 copies/ml] with a fully automated extraction procedure, respectively. To demonstrate clinical application a total of 72 specimens of animal origin were compared with respect to manual and automated extraction. A strong correlation between both methods was observed rendering both methods suitable. Testing of 247 follow up specimens of animal origin from a local Q-fever epidemic rendered real-time PCR more sensitive than conventional PCR.

**Conclusion:**

A sensitive and thoroughly evaluated real-time PCR was established. Its high-throughput mode may show a useful approach to rapidly screen samples in local outbreaks for other organisms relevant for humans or animals. Compared to a conventional PCR assay sensitivity of real-time PCR was higher after testing samples from a local Q-fever outbreak.

## Background

*Coxiella burnetii *(*C. burnetii*) is an obligate intracellular, gram negative bacterium. It is the causative agent of Q-fever. Q-fever is a zoonosis with a worldwide distribution except New Zealand that affects different animal species and humans. Clinical presentation in humans ranges from mild flu-like symptoms to, sometimes, severe atypical pneumonia and hepatitis [1]. Convalescence can be slow and endocarditis is the most frequent and serious manifestation of chronic Q-fever [2]. In animals, primarily cattle, sheep, and goats, *C. burnetii *can cause abortion and infertility as it localizes in the female reproductive system. High doses of *C. burnetii *have been found in conception products of infected animals. The organism is shed in the urine, feces and milk of infected animals. In general infected animals remain asymptomatic. Instead they often serve as the source of infection for humans via infective aerosols or contaminated dust [3]. *C. burnetii *is very resistant to environmental conditions and can remain infectious for a considerable time outside the host cell. Recent outbreaks in France documented the high environmental stability of the organism when local Q-fever epidemics were observed weeks after lambing season [4]. Due to its high infectivity, environmental stability and its potential to cause severe disease in humans it is regarded as a category B biological weapon agent by the Centers for Disease Control and Prevention [5]. Its natural widespread availability and potential for aerosolized use makes it considerably suitable as a biological weapon. Proper administration of antibiotics can significantly reduce chronic Q-fever associated mortality making timely diagnosis of utmost importance. For diagnosing *C. burnetii *infection serology remains the method of choice as it is easy to establish and widely applicable. However, antibodies are detected only after 2–3 weeks from the onset of disease [6] making it too slow in selected clinical settings. A capture enzyme-linked immunosorbent assay (EIA) can be used for direct detection of *C. burnetii *[7]. However, its high limit of detection significantly reduces its reliability. Direct detection of *C. burnetii *is also possible by cell culture, but this requires biosafety level-three laboratories. Sensitivity of cell culture is sometimes low [8]. More recently, PCR has been successfully applied for the direct detection of *C. burnetii *in clinical specimens [9]. Though it appears to be highly sensitive, conventional PCR protocols remain time-consuming due to laborious post PCR processing, and they are prone to cross-contamination. Modern real-time PCR assays with in tube detection of amplicons decrease turn around time considerably [10]. In a bioterrorist event or in the case of local epidemics, masses of samples have to be expected. We have shown for other agents that real-time PCR provides the technical prerequisites for high throughput testing [11].

Here we describe a novel 5'nuclease (TaqMan) based real-time PCR assay for the rapid, sensitive and specific detection of *C. burnetii*. A mimic positive control monitors the reaction under the same conditions as applicable for *C. burnetii*, including use of the same primers. It identifies breaches in sensitivity in each single sample due to insufficient sample preparation, PCR inhibition or inherent failure of the PCR itself. To further facilitate high-throughput application a fully automated extraction procedure using the BioRobot M48 (Qiagen, Hilden, Germany) was evaluated and compared to an established manual sample preparation method.

## Results

### Coxiella burnetii real-time PCR

Since the transposase gene of *C. burnetii *is present in approximately 20 copies per cell it was chosen as the target sequence. Using Primer Express software two primer pairs and one 5'nuclease minor groove binder (MGB) probe were selected. Prepared nucleic acid from a cultured *C. burnetii *(Nine mile RSA493) strain was used to optimize the assay. The 86 bp amplicon of the transposase gene was cloned into *E. coli *plasmids (pCoxquant), and used for sensitivity determination. Single copies were detected in limiting dilution series on an occasional basis. Constantly positive results were obtained at a concentration of 15 copies per PCR reaction or more.

**Figure 1:**
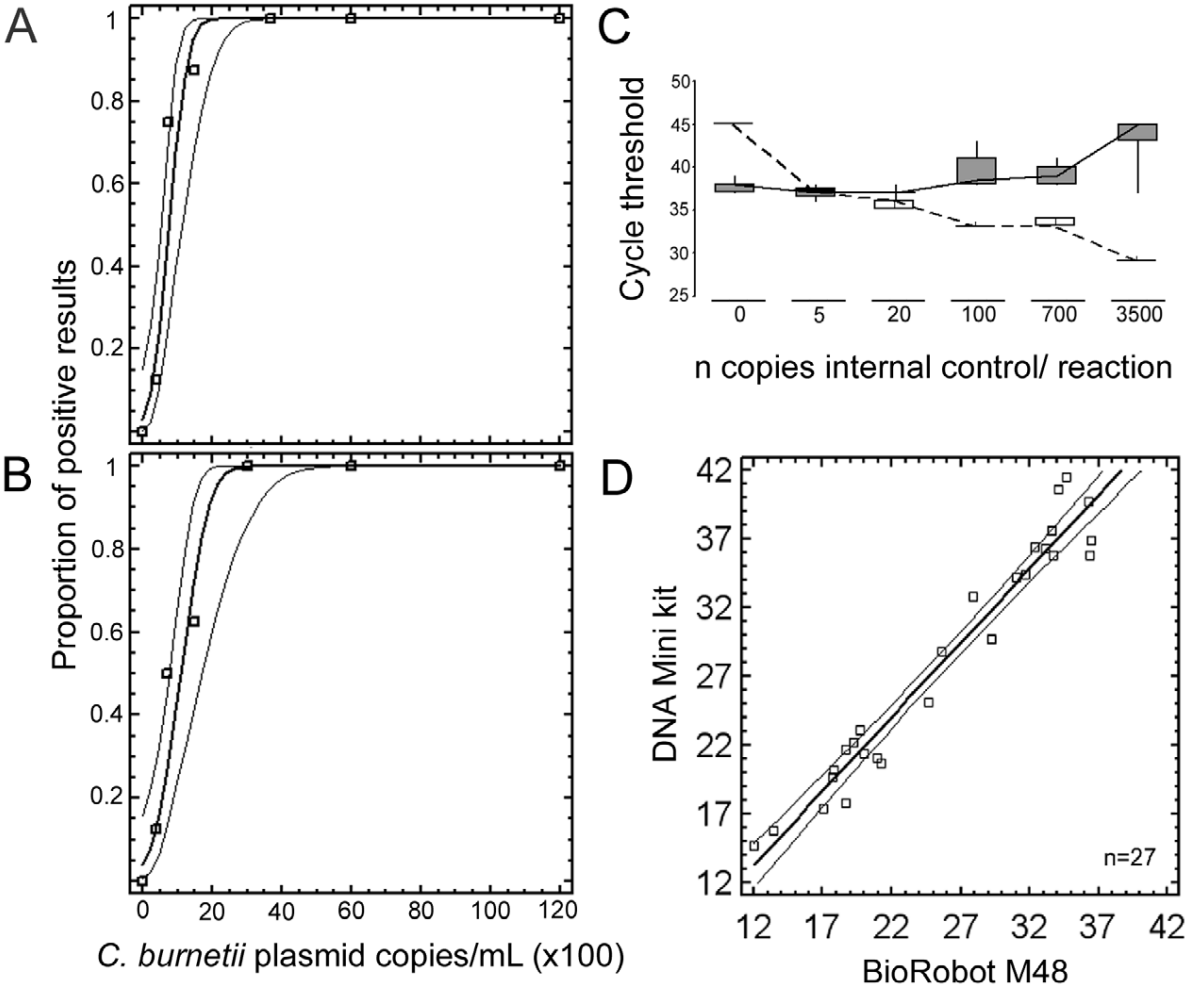
**Determination of detection limits, amplification efficiency of *C. burnetii*, correlation of automated and manual extraction**. Probability of achieving a positive result (y-axis), depending on the DNA input copy number per mL EDTA blood (x-axis). A, Qiagen DNA mini kit; B, Qiagen M48 DNA mini kit, used on a Qiagen M48 automated DNA extraction instrument. Each datum point represents the rate of positive results in six replicate tests per concentration. Limits of detection are comparable with both methods of DNA extraction. C, Threshold cycles (y-axis) as a measure of efficiency of PCR amplification for *C. burnetii *and internal control. Each reaction contained 15 copies of plasmid-derived *C. burnetii *target gene and variable numbers of internal control plasmid pCoxmimic, as depicted on the x-axis. Results of eight replicate real-time PCR reactions per setting are shown as a result of box-plot analysis, showing the range of results by whiskers, whereby the two central quartiles of data are represented as a box. Solid line with grey boxes, *C. burnetii *target gene, broken line with white boxes, internal control. No reduced efficiency in amplification is observed for the *C. burnetii *target gene in presence of up to 100 copies of internal control. D, Correlation of *C. burnetii *DNA copies per ml as determined by *C. burnetii *real-time PCR after automated (x-axis) and manual extraction procedure (y-axis).

To monitor the sensitivity of the assay in each single reaction an internal control mimic DNA was constructed next [12]. The probe binding site of pCoxquant was deleted and replaced by an alternative sequence to yield plasmid pCoxmimic. Its corresponding 5'nuclease probe contained the dye VIC for detection in a separate channel of the real-time PCR instrument. One copy of the internal control mimic pCoxmimic was occasionally and 20 copies constantly detectable. Cross talk into the wild type channel was not observed. To exclude that the internal control affected the amplification efficiency of *C. burnetii *detection, 15 copies/reaction of pCoxquant were amplified in the presence of increasing numbers of pCoxmimic. The cycle threshold (C_t_) values for pCoxquant and pCoxmimic were recorded separately for each target gene (Figure 1C). The simultaneous amplification of up to 100 copies of internal control did not influence the C_t _for pCoxquant. Only from 700 copies of internal control onward, variation and delay in C_t _for pCoxquant occurred. A concentration of 20 copies of pCoxmimic per reaction was chosen as a working concentration for all further experiments, in order to detect even slight drops in assay sensitivity. The exact limit of detection of real-time PCR was determined next. Human EDTA blood was spiked with plasmid pCoxquant in five different concentrations. EDTA blood of each concentration was first extracted manually by means of a QIAmp DNA Mini Kit (Qiagen) in duplicates, and each duplicate was tested in replicates of 4 (5 × 2 × 4 = 40 reactions). Prior to extraction the lysis buffer was spiked with pCoxmimic at a concentration corresponding to 20 copies per reaction. The observed proportions of positive results in each concentration were subjected to probit regression analysis (Figure 1A). 2,881 copies per ml were calculated to be detectable at ≥95% chance (95%CI, 2,188–4,745). This corresponded to 14 copies/PCR reaction.

For automated extraction, the Biorobot M48 (Qiagen) was evaluated exactly in the same manner (Figure 1B). 4,235 (95%CI, 3,143–7,428) copies per ml were calculated to be detectable with ≥95% chance, corresponding to 21 copies per PCR.

The internal control pCoxmimic was detected in all samples including the negative controls. Specificity was evaluated by testing a number of viral and bacterial pathogens that might be present in human blood samples. None of the tested pathogens reacted positive with the *C. burnetii *real-time PCR assay (see Methods section). To further assess the specificity 35 different *C. burnetii *strains were subjected to the new real-time assay. As expected all strains reacted positive (Table 1).

**Table 1:** Characteristics of *C. burnetii *isolates. Designation, geographical origin and host species of *C. burnetii *isolates testing positive by the novel *C. burnetii *real-time PCR

***C. burnetii *strain**	**Geographical origin**	**Host species**
Nine Mile USA	USA	Tick
Priscilla USA	USA	Goat
Scurry USA	USA	Human
Dugway USA	USA	Rodent
Z 2775	Germany	Cattle
Pohlheim	Germany	Sheep
Max	Germany	Sheep
Tiho 1	Germany	unknown
Hardthof/90	Germany	Cattle
Frankfurt	Germany	Cattle
Z 104/94	Germany	Sheep
München	Germany	Sheep
OSH-1	Germany	Cattle
Bru 180	Germany	Cattle
Wdk 1188	Germany	Sheep
Zeckenpool 11	Germany	Tick
Namibia	Namibia	Goat
F-2	France	Human
F-4	France	Human
R1140	Russia	Human
CS-Florian	Slovakia	Human
CS-Bud	Slovakia	Human
CS-KL 4	Slovakia	Tick
CS-Dayer	Slovakia	Tick
Utvinis	Romania	Human
Stancia	Romania	Human
Brasov	Romania	Human
Balaceanu	Romania	Human
J-3	Japan	Cattle
Henzerling	Italy	Human
CS-R	Italy	Human
Herzberg	Greece	Human
Andelfingen	Switzerland	Cattle
Soyta	Switzerland	Cattle
Boren	unknown	Cattle

### Real-time PCR versus conventional PCR

**Figure 2:**
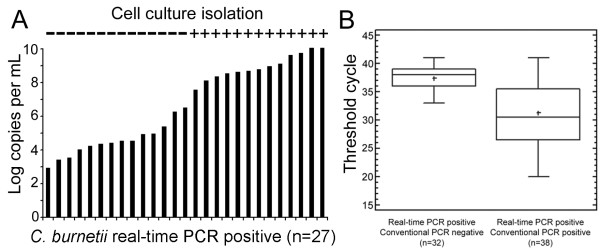
**Bacterial loads in isolation positive samples, box plot analysis of Ct values**. A, bacterial loads and *C. burnetii *isolation in real-time PCR positive samples (n = 27). Bacterial loads are shown on the y-axis. "+" in "cell isolation" means isolation success as confirmed by detection of inclusion bodies upon microscopy. B, box plot analysis of threshold cycle values in real-time PCR positive/conventional negative (n = 32) and real-time PCR positive/conventional PCR positive (n = 38) samples. Difference in threshold cycle values are significant (p < 0.05).

To further assess the performance of real-time PCR we finally compared the assay to a conventional PCR. A total of 247 purified specimens were tested which were sampled during a follow-up study after a local Q-fever epidemic in North-Rhine Westphalia. Results are shown in Table 2 and Figure 2B. The mean C_t _value for samples positive in both assays was 31.31 +/- 2.15 [29.16; 33.46], which equals 2.7 × 10^3 ^copies per PCR (see below). Samples that were positive by real-time PCR only yielded a mean C_t _of 37.42 +/- 0.81 [36.61; 38.23], which equals 48 copies per PCR. Means were significantly different (t-test, p ≤ 0.05).

**Table 2:** Results of real-time versus conventional PCR assay. A total of 247 follow up samples of animal origin were analyzed by conventional as well as real-time PCR. Numbers of positive and negative results for each PCR assay are shown.

	**Real-time PCR positive**	**Real-time PCR negative**	**total**
**PCR positive**	n = 38	n = 5	n = 43
**PCR negative**	n = 32	n = 172	n = 204
**total**	n = 70	n = 177	

### Quantitative real-time PCR

As an example of applicability the novel real-time PCR assay was evaluated in combination with the manual as well as the automated extraction procedure on a panel of samples. Seventy-two homogenized tissue samples were purified using both extraction methods as described above. After purification of samples with the manual extraction method, 27/72 (37.5%) samples yielded a positive result by real-time PCR. With automated extraction 31/72 (43%) samples tested positive. The mean C_t _value in positive samples for each procedure was 28.2 for the manual extraction and 27.65 for the automated procedure, respectively (t-test, p = 0.8028). C_t _values correlated proportionally with DNA concentrations, and showed good accordance between manual and automatic methods (Figure 1D, correlation coefficient = 0.975).

For absolute quantification a calibration curve was generated by limiting dilution series of plasmid pCoxquant. The amplification efficiency calculated from the calibration curve slope was 0.98, indicating optimal PCR reaction condition. Assuming 20 copies of target gene per genome, concentrations of *C. burnetii *per milliliter of homogenized sample ranged from 9.4 × 10^4 ^genomes per mL to 3.2 × 10^10 ^genomes per ml (470-1.6 × 10^8 ^genomes/reaction).

Cell culture has long been regarded the gold standard for diagnosing *C. burnetii *infection. To re-assess its performance, all 72 samples were also subjected to cell culture. Only 13 of 72 (18%) samples yielded a positive result by cell culture. All cell culture positive samples yielded a positive real-time PCR result irrespective of the purification method. Again the bacterial concentration per ml sample was determined and copy numbers of 27 real-time PCR positive samples were plotted in increasing increments and compared to cell culture results (Figure 2A). Only samples with >1 × 10^8 ^genomes/ml of homogenized sample yielded a positive result by cell culture.

## Discussion

Recent experience has shown that in the event of a *C. burnetii *epidemic masses of samples are to be expected [13]. We describe here a *C. burnetii *real-time PCR assay in combination with manual as well as fully automated extraction procedures for high-throughput use. The assay is capable of detecting single copies of *C. burnetii *transposase gene. This target region was chosen because it is highly conserved among *C. burnetii *strains and present at 10–30 copies per bacterium, making it an appropriate target for a diagnostic assay [14-16]. However, despite the assay was validated on a range of *C. burnetii *isolates, strains lacking elements of the transposase gene have been described [17]. For our calculations of sensitivity we assumed 20 copies per genome as present in our Nine Mile RSA 493 reference strain [16]. At 2,881 DNA copies/ml blood, the analytical sensitivity of our assay was close to what is possible due to mathematical limitations, according to the probit model [18]. This sensitivity, corresponding to about 144 genomes per ml, was as high as with commercial PCR systems available for other bacterial agents and should be compatible with the reliable detection of bacteremia [19]. Of note, since the number of copies of the *C. burnetii *transposase gene can vary greatly different sensitivities have to be expected with other strains.

Due to increased hybridization properties and lower background fluorescence we decided to use a minor groove binder (MGB) probe in combination with a non-fluorescent-quencher (NFQ) [20]. The use of a NFQ improves the signal-to-noise ratio and decreases spectral overlap compared to other fluorescent quencher dyes e.g. TAMRA [21]. This feature facilitates double dye detection, which allows implementation of a stable internal control system. The internal control was detected in all negative samples irrespective of the extraction procedure, although only a low amount of internal control mimic was added (20 copies plasmid DNA per reaction). Since the internal control is almost identical to the *C. burnetii *target gene including additional positive controls is not necessary. The advantages of such controls have already been described and implemented in various assays [22,23].

Besides the PCR itself, the extraction procedure constitutes another crucial factor in molecular diagnostics. It is the lengthiest part of the whole procedure and automation is highly desirable especially in a high-throughput setting. The most valuable advantage of robotic systems is their speed when numerous samples have to be analyzed [24]. However, there is little data on the performance of automated systems in diagnostic settings. In this study the limit of detection of the automated procedure was only 1.5 times higher than that of the manual protocol (4,235 vs. 2,881 DNA copies/ml blood). Assuming 20 copies per bacterium (Nine mile RSA493 strain) the limit of detection for the automated procedure was around 210 *C. burnetii *bacteria per ml (1 bacterium per PCR reaction). High sensitivity and good quantitative correlation with other extraction methods is in concordance with our earlier study on the automated extraction of *B. anthracis *[11].

The novel assay yielded more positive results than conventional PCR upon testing of a large panel of stored samples of animal origin. However, it should be noted that 2.5 times more template was added to the real-time PCR reaction. Because PCR negative/real-time PCR positive samples had significantly lower C_t _values than samples positive in both methods, improved analytical sensitivity can be assumed for real-time PCR. Of note 5 conventional PCR positive samples remained repeatedly negative upon real-time PCR testing. All of these 5 samples were stored for approximately 3 years at -20°C and reported to be weakly positive only by conventional PCR. Due to prolonged storage it seems likely that some DNA degradation had happened thus preventing positive results by real-time PCR.

Critically, classical isolation of *C. burnetii *by cell culture showed rather disappointing results. Henning and Sting have already reported similar results for cell culture [25]. The strain of *C. burnetii *as well as its source might account for the here observed rather low number of conventional cell culture positive results. More sensitive isolation methods like shell vial technique or inoculation of specimens into guinea pigs or mice were not available in our laboratory. By quantitative PCR we could show that rather high bacterial loads are necessary for cell culture to become positive. For this reason and because of the associated biological risks, cell culture is not the method of choice in the first run. However, it remains an essential tool for further pathogen characterization, and allows isolation of related agents which would go undetected by PCR.

## Conclusion

In summary, this assay provides a homogenous tool for diagnostics in human and veterinary medicine. Since *C. burnetii *infection is still not well understood, quantitative PCR may yield new insights into the pathogenesis of the disease. Another future field of application of high throughput assays may comprise the screening of bulk milk samples, an issue which may be addressed by food safety authorities.

## Methods

### Reference strain

A cultured *C. burnetii *(Nine mile RSA493) strain was kindly donated by D. Raoult, Unite de Rickettsies, Universite de la Mediterranee, Marseille, France.

### Samples

To evaluate two different extraction procedures 53 specimens were tested, which were sampled during a local Q-fever epidemic among sheep in North-Rhine Westphalia/Germany in 2003. All samples were placenta material. In addition 19 specimens collected independently by the Friedrich-Loeffler-Institute (FLI) from different other sources were analyzed. For specificity testing a panel of 35 *C. burnetii *strains was tested. In addition, 27 different bacteria and viruses which might be present in clinical samples were obtained from the American Type Culture Collection (Manassas, VA, USA), the German Collection of Microorganisms and Cell Cultures (DSMZ, Braunschweig, Germany) or our in house strain collection. For an additional part of the study 247 aliquots of previously purified DNA samples from a follow-up examination of placenta material after the local epidemic of Q-fever in North-Rhine Westphalia were available.

### Cell culture

Isolation of *C. burnetii *was performed using Buffalo Green Monkey (BGM) cells. Cells were propagated in 25 cm^2 ^plastic flasks with in UltraCulture medium (BioWitthaker, Walkersville, Maryland, USA) without supplements. Material of each sample (1 g) was homogenised using sterile mortar, sand, and cell culture medium. The supernatants were filtered through membrane filters (Minisart™ Sartorius, Göttingen, Germany) with pore diameters of 0.2 μm. A volume of 0.5 ml homogenate per flask was inoculated. Cell cultures were examined weekly by phase-contrast microscopy for inclusion bodies.

### Sample preparation

Samples were first homogenized for cell culture as described above. For conventional PCR assays nucleic acids were prepared using the Puregene DNA Blood Isolation Kit (Gentra Systems, Minneapolis, USA). One mL of the homogenized sample was centrifuged at 12,000 g for 10 min. The pellet was resuspended in 600 μl lysis buffer and incubated at 80°C for 5 min. Further steps were as recommended by the manufacturer.

### Conventional PCR

A conventional PCR with primers amplifying a 448 bp product of the transposase gene of *C. burnetii *(IS1111) was conducted. Amplification was carried out in a total reaction volume of 50 μl with 1× PCR Buffer, 2 mM MgCl_2_, 200 μM of each dNTP and 0.5 units of Taq Polymerase (Bioline, Luckenwalde, Germany), 0.5 μM of primer CoxP4 (ttaaggtgggctgcgtggtgatgg, nt positions 222–245 in GenBank accession M80806) (TIB-Molbiol, Berlin, Germany), 0.5 μM of primer CoxM9 (gcttcgtcccggttcaacaattgc, nt 669–648) (TIB-Molbiol) and 2 μl of total DNA. Thermal cycling involved 94°C for 9 min, followed by 5 cycles of 94°C 30 s, 75°C to 67°C 30 s with 2 °C decrements per cycle, 77°C 30 s; and 37 cycles of 94°C 30 s, 65°C 30 s, 77°C 30 s with a final elongation step at 77°C 2 min. PCR products were visualized by gel electrophoresis on a standard 1% agarose gel stained with ethidium bromide.

### Sample preparation/real-time PCR assay

Total genomic DNA was extracted manually using the QIAmp DNA Minikit (Qiagen, Hilden, Germany) from aliquots of homogenized samples as described above. One hundred microliters of sample was added to 100 μl of buffer ATL (Qiagen). Twenty microliters of Qiagen Proteinase K were added, and incubated at 56°C for 30 min. Next 200 μl of buffer AL (Qiagen) was added, which was previously spiked with the *C. burnetii *internal control plasmid at a final concentration of 20 copies per PCR reaction. Further recommendations of the manufacturer were followed (200 μl elution volume). Alternatively, genomic DNA was extracted by an automated procedure using the BioRobot M48 (Qiagen) in combination with a MagAttract DNA Mini Kit (Qiagen). One hundred microliters of sample were added to 100 μl buffer G2 (Qiagen) to which the internal control was previously added at a concentration corresponding to 20 copies per PCR reaction. Ten microliters of Proteinase K solution (Qiagen) were added and incubated at 56°C for 30 min. Further procedure was as recommended by the manufacturer, setting the elution volume to 200 μl.

### Plasmid standard (pCoxquant)

An 86 bp insert of the transposase gene of *C. burnetii *was ligated into plasmid vectors and cloned in *E. coli *by means of a pCR2.1-TOPO TA cloning kit (Invitrogen, Karlsruhe, Germany). Plasmids were purified with a NucleoSpin Plasmid kit (Macherey Nagel, Düren, Germany) and sequenced using the BigDye 3.1 terminator cycle sequencing chemistry (Applied Biosystems, Weiterstadt, Germany) on an automatic ABI 377 DNA sequencer (Applied Biosystems). Plasmid DNA content was measured spectrophotometrically.

### Internal control plasmid (pCoxmimic)

The *C. burnetii *plasmid pCoxquant was used to generate an internal control plasmid. Gene splicing by overlap extension was carried out using primers IcS (atcgttcgttgagcgattagcagttgccaatttaaatcgtgatgccggat) and IcAs (aactgctaatcgctcaacgaacgatgcaaggttgatgcttatcgggctatc) to introduce an alternative probe-binding site at nucleotide positions 1266–1293 (GenBank accession M80806). Resulting constructs of the correct length were cloned by means of a pCR 2.1-TOPO TA cloning kit (Invitrogen) and processed as described above.

### Oligonucleotide design

Using primer Express software with default settings for 5'nuclease (TaqMan) minor groove binder (MGB) probes (Applied Biosystems) two possible primer combinations and one probe were evaluated. GenBank accession M80806 served as the query sequence. The optimal primer/probe combination was experimentally determined by checkerboard titration first. In a next set of experiments individual primer concentration was optimized and finally the magnesium concentration adjusted.

### Real-time 5'nuclease PCR

A 25 μl reaction volume contained 5 μl of DNA, 4 mM MgCl_2_, 1× Platinum Taq polymerase reaction buffer (Invitrogen), 200 μM of each dNTP, 0.8 μM primer CoxbS (gatagcccgataagcatcaac, nt position 1241–1261, GenBank accession M80806) (TIB-Molbiol), 0.8 μM primer CoxbAs (gcattcgtatatccggcatc, nt 1326–1307) (TIB-Molbiol), 0.3 μM probe CoxbMGB (tcatcaaggcaccaat, nt 1272–1287), 0.2 μM probe prCoxmutant (atcgttcgttgagcgattagcagtt) and 1 unit of Taq DNA polymerase. 5'nuclease probe CoxbMGB was labeled with 5'FAM and a 3' minor groove binder non-fluorescent quencher (Applied Biosystems), 5'nuclease probe prCoxmutant was labeled with 5'VIC and 3'Black Hole Quencher (Eurogentec, Seraing, Belgium). Cycling conditions in an ABI Prism 7000 machine (Applied Biosystems) were: 95°C/2 min, and 45 cycles of 95°C/15 sec, 60°C/30 sec. Data were analyzed with the Sequence detector software V 2.1 (Applied Biosystems).

### Specificity panel

*Bacillus cereus *(ATCC 4313), *Bacillus subtilis *(ATCC 6633), *Candida albicans *(ATCC 10231), *Chlamydophila pneumoniae *(CWL-029H), *Enterococcus faecalis *(in house reference strain), Epstein-Barr virus (patient isolate), *Escherichia coli *(ATCC 25922), Hepatitis B virus (INSTAND ref. material 11019), Hepatitis C virus (1^st ^International WHO NAT standard), Human cytomegalovirus (INSTAND ref. material 15005), Herpes simplex virus type 1 (INSTAND ref. material 13017), Human immunodefieciency virus type 1 (NL-43), *Mycobacterium tuberculosis *(in house reference strain), *Orientia tsutsugamushi *(in house reference strain), *Plasmodium falciparum *(patient isolate), *Pseudomonas aerogenosa *(in-house reference strain), *Rickettsia conorii *(in house reference strain), *Rickettsia prowazeckii *(in house reference strain), *Rickettsia rickettsii *(in house reference strain), *Salmonella enteritidis *(in house reference strain), *Shigella sonnei *(in house reference strain), *Staphylococcus aureaus *(ATCC 13565), *Streptococcus pneumoniae *(ATCC 6305), *Streptococcus pyogenes *(ATCC 19615)

### Statistical analysis

Different input concentrations of *C. burnetii *plasmid DNA pCoxquant were tested to calculate the predicted proportion of positive results in replicate tests using probit analysis as a non-linear regression model. Statgraphics plus version 5.1 software was used for probit analysis (Manugistics, Dresden, Germany).

## Authors' contributions

MP established the real-time PCR assay, coordinated the study and drafted the manuscript. JK, SG-F and MPE prepared and analyzed samples of the follow up examination. SK participated in performing real-time PCR experiments and provided the cross reactivity panel. DF and HM provided the specificity panel, performed experiments and drafted the manuscript. KH was responsible for cultivation of *C. burnetii *isolates and helped to design the study. CD participated in the design of the study, provided technical and financial support and helped to draft the manuscript. All authors read and approved the final manuscript.
